# Copper ionophore elicits calpain-dependent paraptosis coincident with proteotoxic stress

**DOI:** 10.1186/s12964-025-02558-5

**Published:** 2025-12-12

**Authors:** Apiwit Sae-Fung, Bengt Fadeel

**Affiliations:** 1 Division of Molecular Toxicology, Institute of Environmental Medicine, Karolinska Institutet, Stockholm, Sweden; 2 Graduate Program in Clinical Biochemistry and Molecular Medicine, Department of Clinical Chemistry, Faculty of Allied Health Sciences, Chulalongkorn University, Bangkok, Thailand

**Keywords:** Calpain, Cell death, Copper, Heat shock response, Proteasome, Proteomics

## Abstract

Copper is essential to all living organisms. However, too much copper is deleterious, and cellular copper content is therefore subject to tight control. Excess copper was recently found to perturb a set of metabolic enzymes in mitochondria, leading to the aggregation of these proteins and the demise of the cell. However, our understanding of the mechanism of copper-dependent cell death remains incomplete. Here, we report that copper ionophore (elesclomol)-induced cell death is calpain-dependent, featuring dilation of the endoplasmic reticulum along with perinuclear clustering of mitochondria. Moreover, elesclomol evoked proteotoxic stress, manifested as a disruption of ubiquitin and proteasome homeostasis, coupled with a conserved heat shock response. Overall, these results have shown that elesclomol promotes calpain-dependent paraptosis with the involvement of both mitochondrial and extramitochondrial compartments of the cell.

**Graphical abstract:**

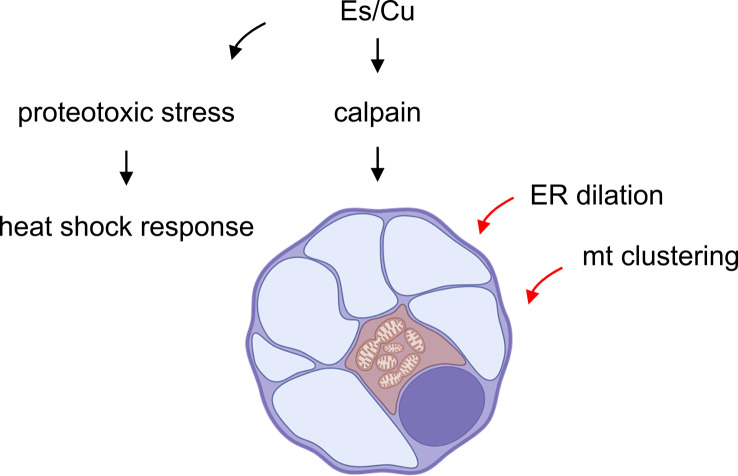

**Supplementary Information:**

The online version contains supplementary material available at 10.1186/s12964-025-02558-5.

## Introduction

Copper is a double-edged sword: it is an essential micronutrient, yet deregulation of cellular copper status can lead to cell death and/or copper-dependent cell proliferation (cuproplasia) [1]. Indeed, cellular copper status may be exploited for therapeutic gain, and both copper ionophores and copper chelators have been suggested as potential anticancer agents [1]. Recent work demonstrated that cancer cells that rely on mitochondrial metabolism rather than glycolysis undergo cell death in response to the copper ionophore elesclomol, and a genome-wide screening approach showed that the gene encoding the small iron sulfur (Fe–S) cluster protein, ferredoxin 1 (FDX1), could rescue this cell death [2]. Mitochondrial ferredoxins were previously identified as relevant copper targets in yeast [3]. Subsequent work confirmed the link between mitochondrial metabolism and copper-dependent death (cuproptosis) of cancer cells, and it was demonstrated that excess copper promotes the aggregation of lipoylated proteins belonging to the tricarboxylic acid (TCA) cycle in mitochondria [4]. Other investigators provided independent evidence that lipoylation is a target of elesclomol [5].

Cuproptosis, as it is currently understood, i.e., a non-apoptotic cell death evoked by a copper ionophore that targets copper to mitochondria [6], leading to the aggregation of lipoylated proteins [2, 4], has received considerable attention, as the prospect of selectively culling cancer cells holds great promise [1]. However, the mechanism through which elesclomol triggers cell death is not completely resolved, and the morphological attributes of this novel cell death have not yet been defined [7]. Moreover, it is conceivable that other copper-induced cell death modalities exist, and more work is therefore needed to delineate the spectrum of cuproptosis or cuproptosis-like forms of cell death [8]. To this end, we embarked on a study of copper-induced cell death using elesclomol. The human fibrosarcoma cell line HT-1080, a workhorse of cell death research [9], was selected as a model along with its normal fibroblast counterpart (BJ) [10]. Our findings show that Es/Cu-induced cell death is calpain-dependent, and evidence is provided both for mitochondrial and extramitochondrial involvement in this paradigm.

## Results

### Elesclomol induces cell death with features of paraptosis

**Fig. 1:**
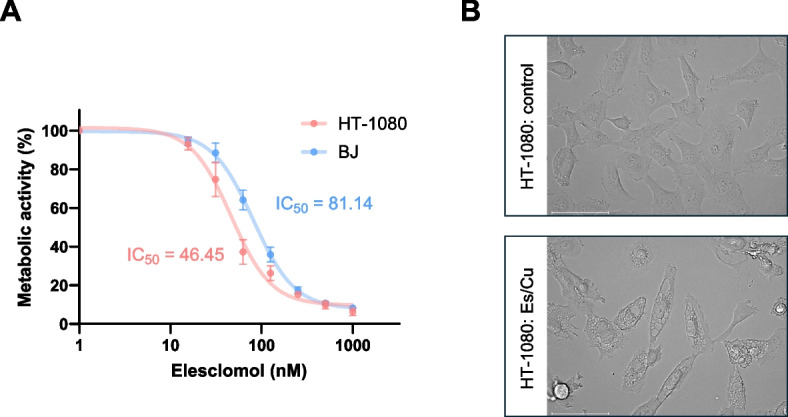
Elesclomol triggers cell death with cytoplasmic vacuolization. **A** Viability of HT-1080 and BJ cells exposed to Es/Cu for 24 h. Different concentrations of Es were evaluated in the presence of CuCl_2_ (1 µM). The IC_50_ values are indicated. Data are shown as mean values ± S.D. (*n* = 3). **B** HT-1080 cells exposed to medium alone or to Es/Cu (50 nM) for 24 h were visualized under the light microscope. Scale bars: 75 µm

**Fig. 2:**
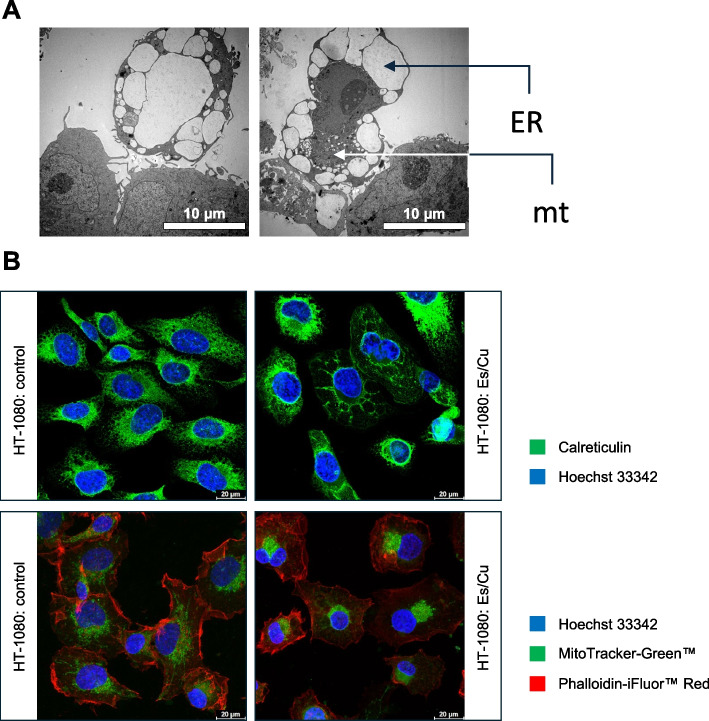
Elesclomol causes ER dilation and perinuclear clustering of mitochondria. **A** TEM images of HT-1080 cells exposed to Es/Cu (50 nM) for 16 h. ER, endoplasmic reticulum; mt, mitochondria. Scale bars: 10 µm. **B** Confocal images of HT-1080 cells exposed to medium alone or to Es/Cu (50 nM) for 24 h, stained with a calreticulin-specific antibody (ER marker) followed by a secondary antibody conjugated to Alexa Fluor™ 488 (green) (upper panels), or stained with MitoTracker™ Green for mitochondria and Phalloidin-iFluor™ Red for F-actin (red) (lower panels). Cell nuclei were counterstained in both cases by using Hoechst 33342 (blue). Scale bars: 20 µm

**Fig. 3:**
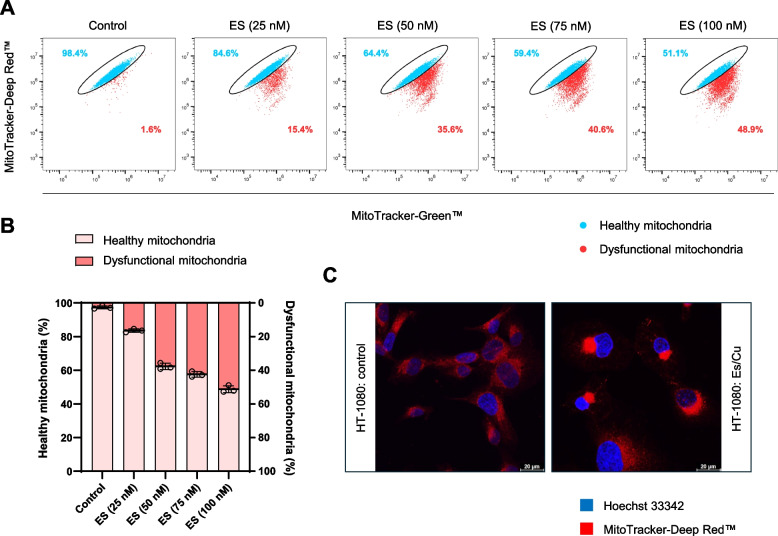
Elesclomol induces dose-dependent mitochondrial dysfunction. **A** Flow cytometric analysis of HT-1080 cells exposed for 24 h to Es/Cu at the indicated concentrations and stained with MitoTracker™ Deep Red and MitoTracker™ Green. **B** Bar charts showing the percentage of healthy *versus* dysfunctional mitochondria. Data shown are mean values ± S.D. (*n* = 3). **C** Confocal images of HT-1080 cells exposed to medium alone or to Es/Cu (50 nM) for 24 h, stained with MitoTracker™ Deep Red for mitochondria and Hoechst 33342 for cell nuclei (blue). Scale bars: 20 µm

To address whether copper-loaded Es (hereafter: Es/Cu) (Figure S1) triggers cell death under normal growth conditions, HT-1080 cells cultured in glucose-containing medium were exposed to Es/Cu. Dose-dependent cell death was observed using the LDH release assay (Figure S2A). The BJ fibroblast cell line responded in a similar manner (Figure S2B). Using alamarBlue™, a fluorometric indicator of cell metabolic activity, we found that the half maximal inhibitory concentration (IC_50_) was lower for HT-1080, indicating that fibrosarcoma cells are more susceptible than fibroblasts (Fig. 1A). On the other hand, CuCl_2_ alone failed to trigger cell death (Figure S3). Cytoplasmic vacuolization was observed in cells undergoing Es/Cu-induced cell death (Fig. 1B). We therefore subjected HT-1080 cells to transmission electron microscopy (TEM) to gain a better understanding of these morphological changes. Indeed, prominent cytoplasmic vacuolization was observed, reminiscent of paraptosis [11] (Fig. 2A). We also noted the absence of chromatin condensation (a characteristic feature of apoptosis) [12] while mitochondria appeared to congregate in a unipolar fashion in cells exposed to Es/Cu. Confocal microscopy of cells stained with antibodies against calreticulin showed that the vacuoles were derived from the endoplasmic reticulum (ER) (Fig. 2B). Furthermore, perinuclear clustering of mitochondria was demonstrated by using a mitochondria-specific fluorescent dye along with counterstaining of the cell nuclei (Fig. 2B). To evaluate mitochondrial ‘fitness’, we performed flow cytometry using two fluorescent dyes to distinguish mitochondrial respiration (as evidenced by mitochondrial transmembrane potential) (MitoTracker-Deep Red™) *versus *mitochondrial mass (MitoTracker-Green™) [13]. Evidence was obtained for a dose-dependent induction of dysfunctional mitochondria (Fig. 3A,B), and perinuclear clustering of mitochondria could also be confirmed (Fig. 3C). Thus, Es/Cu exposure evokes cell death with involvement of both mitochondria and the ER. In parallel experiments, the HT-1080 cell line was shown to be apoptosis competent as etoposide triggered caspase-dependent cell death (data not shown). HT-1080 cells were also found to undergo etoposide-induced apoptosis by other investigators [14]. Hence, the results obtained for Es/Cu are not explained by a malfunctioning apoptosis machinery.

**Fig. 4:**
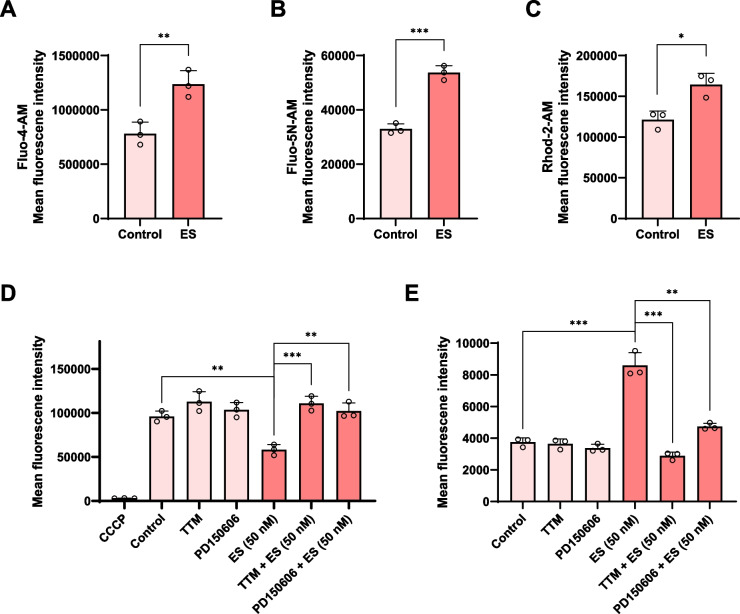
Elesclomol triggers calcium elevation and ROS production. **A-C** Flow cytometric analysis of HT-1080 cells exposed to Es/Cu (50 nM) for 16 h. Fluo-4 AM and Fluo-5N AM were used for the detection of low and high cellular calcium levels, respectively, while the Rhod-2 AM indicator was used to detect mitochondrial calcium levels. The dissipation of the mitochondrial transmembrane potential was determined using TMRE (**D**), and the mitochondrial uncoupling agent CCCP (5 μM) was included as a positive control, while mitochondrial superoxide levels were measured using MitoSOX™ (**E**). The cells were exposed to Es/Cu (50 nM) for 16 h in the presence or absence of the copper chelating agent, TTM (10 µM) or the selective calpain inhibitor, PD150606 (100 µM). Data are shown as mean values ± S.D. (*n* = 3). *p < 0.05; **p < 0.01; ***p < 0.001

Using a panel of calcium indicators, we could show that Es/Cu also evoked a significant elevation of intracellular calcium (Fig. 4A-C). Fluo-4 AM is a cell permeable high-affinity calcium indicator while its analog Fluo-5N AM is a low-affinity calcium indicator suitable for monitoring high calcium levels in, for example, the ER. The cationic Rhod-2 AM indicator, in turn, is used to monitor mitochondrial calcium levels [15]. Es/Cu triggered the dissipation of the mitochondrial transmembrane potential along with mitochondrial superoxide production (Fig. 4D,E). These effects were reversed by the copper chelating agent, TTM (tetrathiomolybdate) and by the selective calpain inhibitor, PD150606. The role of calpain is explored in detail in the following sections.

### Copper-loaded elesclomol elicits non-apoptotic cell death

**Fig. 5:**
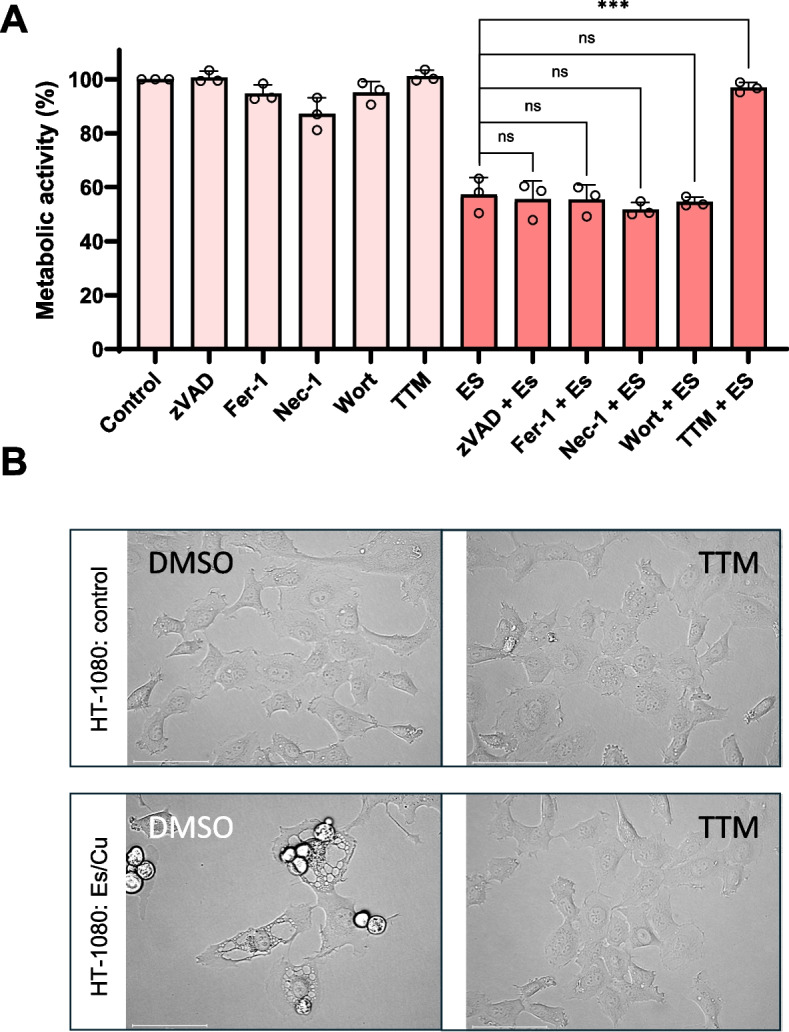
Copper chelation protects against elesclomol-induced cell death. **A** HT-1080 cells were exposed to Es/Cu (50 nM) for 24 h in the presence or absence of the apoptosis inhibitor, zVAD-fmk (20 µM), ferroptosis inhibitor, ferrostatin-1 (10 µM), necroptosis inhibitor, necrostatin-1 (20 µM), autophagy (phosphatidylinositol 3-kinase) inhibitor, wortmannin (2 µM), or the copper chelator, TTM (10 µM). Data are shown as mean values ± S.D. (*n *= 3). ns, not significant; ***p < 0.001. **B** HT-1080 cells were exposed to vehicle alone (DMSO) or to Es/Cu (50 nM) for 24 h in the presence or absence of TTM (10 µM) and visualized under the light microscope. Scale bars: 75 µm

To understand whether Es/Cu triggers a known cell death mechanism, cells were preincubated with inhibitors of apoptosis (zVAD-fmk), ferroptosis (ferrostatin-1), necroptosis (necrostatin-1), and autophagy (wortmannin). However, all failed to rescue HT-1080 cells from Es/Cu-induced cell death (Fig. 5A). On the other hand, the copper chelating agent, TTM completely reversed cell death, proving that cell death was due to an overload of copper, and prevented the cytoplasmic vacuolization induced by Es/Cu (Fig. 5A,B).

FDX1 was recently identified as a critical regulator of cuproptosis [2, 4]. To study the role of FDX1 in our model, we generated cells with specific silencing of *FDX1 *using shRNA (Figure S4). These were then exposed for 24 h, 48 h, or 72 h to Es/Cu. However, loss of FDX1 expression did not impact the metabolic capacity of the cells regardless of whether the cells were cultured in glucose- or galactose-containing medium (Figure S5A-F). We then asked whether STEAP3, an endosomal ferrireductase that also reduces copper [16], is involved in the regulation of Es/Cu-induced cell death. However, silencing of *STEAP3* in HT-1080 cells also failed to block cell death (data not shown).

### Elesclomol-induced cell death is mediated by calpain activation

**Fig. 6:**
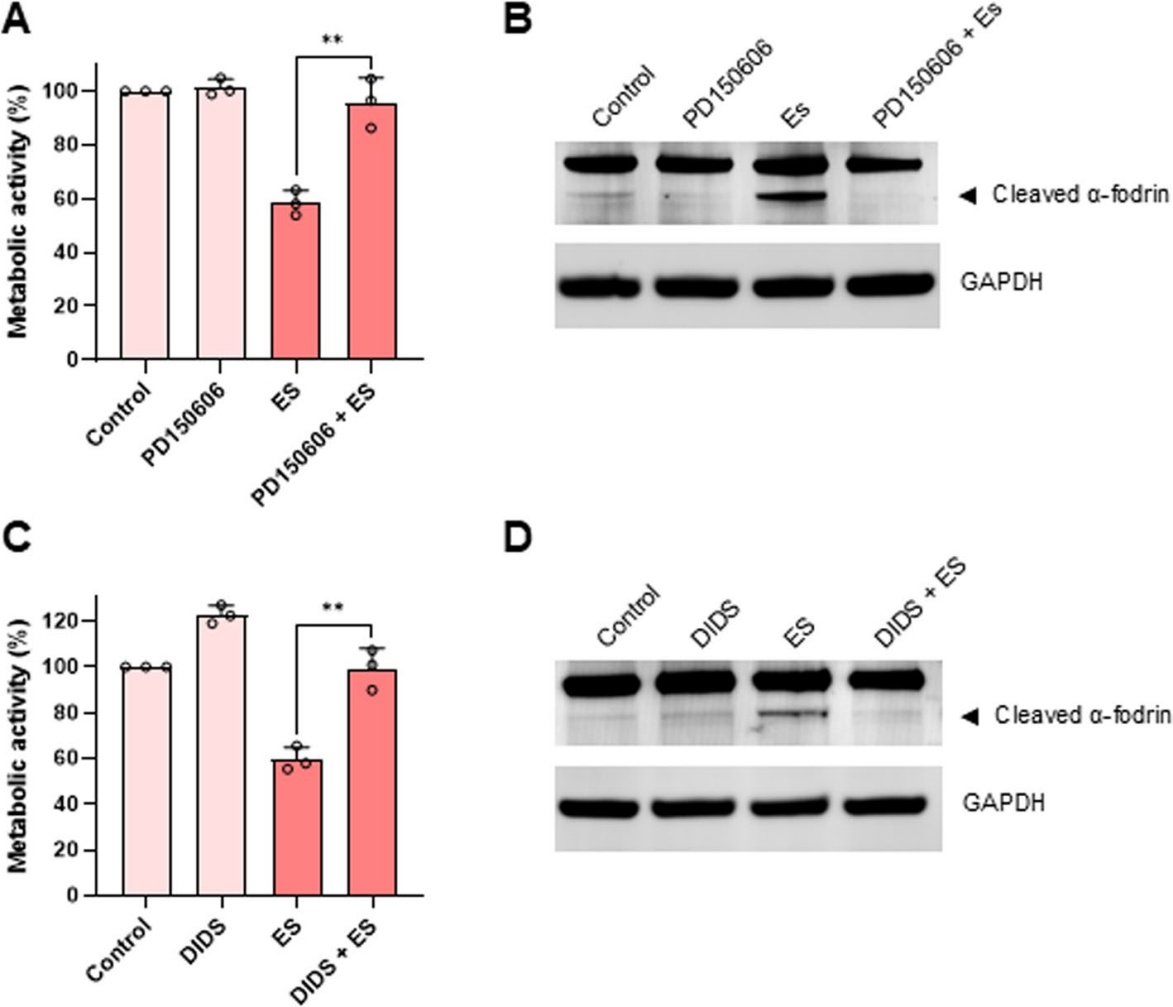
Elesclomol-induced cell death is calpain-dependent. **A**, **C** Viability of HT-1080 cells exposed to Es/Cu (50 nM) in the presence or absence of the calpain inhibitor, PD150606 (100 µM) or the non-selective inhibitor of VDAC oligomerization, DIDS (200 µM). Data shown are mean values ± S.D. (*n* = 3). **p < 0.01. **B**, **D** Western blot of α-fodrin cleavage in HT-1080 cells exposed to Es/Cu (50 nM) in the presence or absence of PD150606 (100 µM) or DIDS (200 µM). GAPDH was included as a loading control

**Fig. 7:**
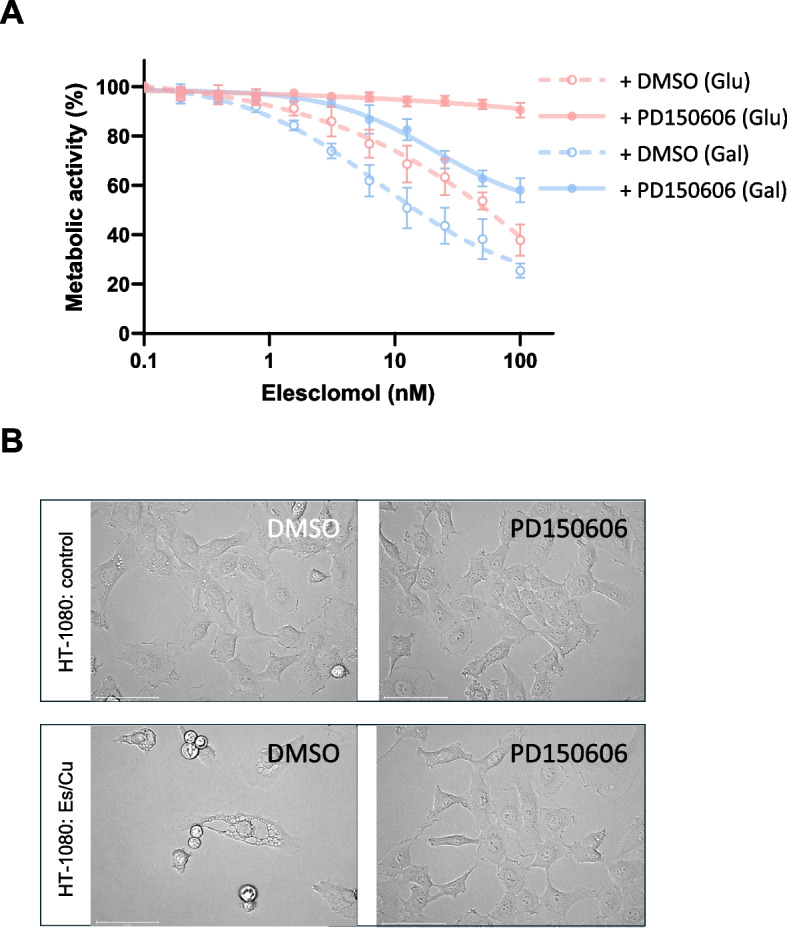
Impact of metabolic status on elesclomol-induced cell death. **A** Viability of HT-1080 cells exposed to Es/Cu in the presence or absence of the calpain inhibitor, PD150606 (100 µM) in cell medium containing glucose (pink) or galactose (blue). Data are shown as mean values ± S.D. (*n* = 3). **B** HT-1080 cells were exposed for 24 h to vehicle alone (DMSO) or to Es/Cu (50 nM) with or without PD150606 (100 µM) in glucose-containing medium and visualized under the light microscope. Scale bars: 75 µm

Previous work provided evidence for calpain activation in HT-1080 cells exposed to ꝩ-radiation [17]. Moreover, we demonstrated that non-apoptotic (caspase-independent) cell death of the promyelocytic HL-60 cell line is regulated by calpain [18]. To test the idea that Es/Cu-induced cell death is calpain-dependent, HT-1080 cells were pre-incubated with the selective calpain inhibitor, PD150606. Es/Cu-induced cell death was abolished upon calpain inhibition, and cleavage of α-fodrin, a known calpain substrate, was also blocked (Fig. 6A,B). Furthermore, PD150606 rescued the BJ fibroblast cell line from cell death (Figure S6A,B). Cuproptosis depends on mitochondrial metabolism [8]. To evaluate whether or not mitochondrial metabolism might impinge on Es/Cu-induced cell death in our model, cells were maintained in cell medium with glucose or galactose. HT-1080 cells cultured in glucose-containing medium succumbed to Es/Cu-induced cell death and were rescued by PD150606, and cytoplasmic vacuolization was also abolished in the presence of the calpain inhibitor (Fig. 7A,B). Cells maintained in cell medium with galactose to foster mitochondrial metabolism were more sensitive to Es/Cu, as could be expected, and cell death was suppressed, but not completely blocked, by the calpain inhibitor, PD150606 (Fig. 7A). The calpain(s), non-lysosomal cysteine proteases, thus emerge as potential regulators of copper ionophore-induced cell death.

Cytoplasmic vacuolization in paraptosis may be related to the distension of mitochondria as well as the ER [19]. Moreover, mitochondria and ER are engaged in bidirectional communication through physical contact sites referred to as mitochondria-associated membranes or MAMs [20]. Numerous regulatory proteins reside at the MAMs. To explore the role of MAMs, we screened several inhibitors of proteins present in MAMs. We found that DIDS (4,4'-diisothiocyano-2,2'-dihydrostilbenedisulfonic acid) rescued HT-1080 cells from Es/Cu-induced cell death, potentially upstream of calpain, as cleavage of α-fodrin was also blocked (Fig. 6C,D). DIDS is thought to prevent the oligomerization of VDAC1 [21], although it cannot be considered a specific VDAC1 inhibitor given its interactions with various other transporters. Nevertheless, our findings suggest that VDAC1 may be involved in Es/Cu-induced paraptosis. In contrast, the inositol trisphosphate (IP_3_) receptor antagonist 2-APB and the sarcoplasmic/endoplasmic reticulum calcium-ATPase (SERCA) inhibitor, thapsigargin did not affect Es/Cu-induced cell death (Figure S7). VDAC1 serves as a conduit for calcium but is also involved in cell metabolism by shuttling ATP and other metabolites [22].

### Glutathione and NAC protect against copper-induced cell death

**Fig. 8:**
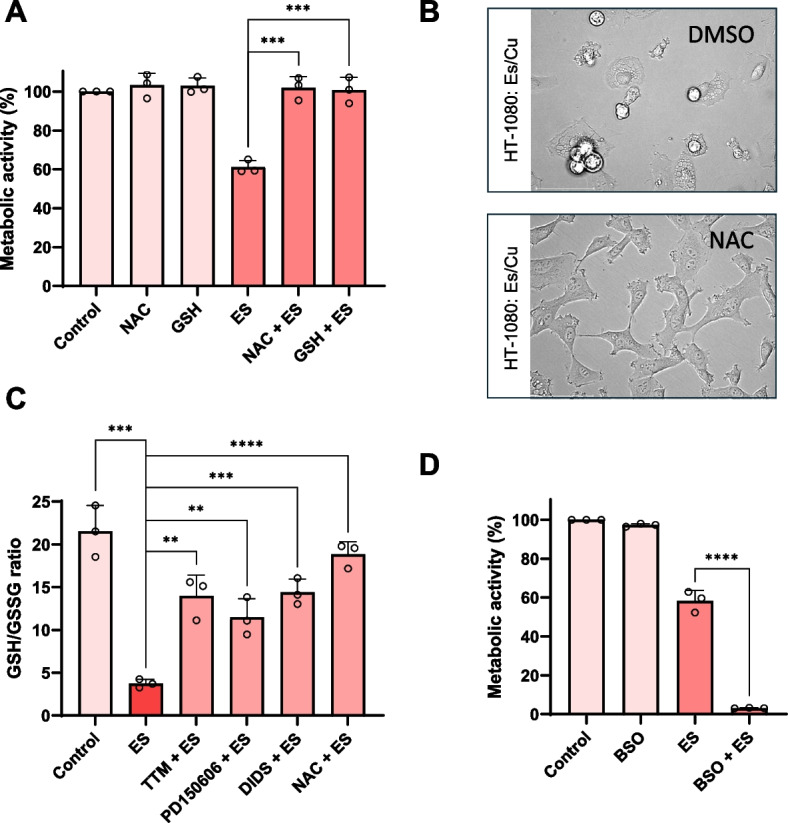
GSH and NAC protect against elesclomol-induced cell death. **A** Viability of HT-1080 cells exposed to Es/Cu (50 nM) for 24 h in the presence or absence of NAC (10 mM) or GSH (5 mM). **B** HT-1080 cells were exposed to Es/Cu (50 nM) for 24 h in the presence of NAC (10 mM) or vehicle alone (DMSO) and visualized under the light microscope. Scale bars: 75 µm. **C** GSH/GSSG ratio in HT-1080 cells exposed to Es/Cu (50 nM) for 16 h in the presence or absence of the copper chelator, TTM (10 µM), calpain inhibitor, PD150606 (100 µM), VDAC inhibitor, DIDS (200 µM), or NAC (10 mM). **D** Viability of HT-1080 cells exposed to Es/Cu (50 nM) for 24 h in the presence or absence of BSO (10 µM), an inhibitor of GSH synthesis. Data in panels (**A**), (**C**), and (**D**) are mean values ± S.D. (*n* = 3). **p < 0.01; ***p < 0.001; ****p < 0.0001

N-acetylcysteine (NAC), a precursor of cysteine and, therefore, glutathione (GSH), is an antioxidant. However, GSH also functions as an intracellular copper chelator [23]. In fact, a recent study demonstrated that the protective role of GSH against copper overload is based on the binding of Cu(I) ions rather than on the antioxidant properties of GSH [24]. On the other hand, the authors found that NAC (at concentrations below 5 mM) failed to rescue the cells. Similarly, Tsvetkov et al. found that NAC (1 mM) failed to block cuproptosis. [4] In contrast, other investigators suggested that it is the reduction in cellular copper uptake rather than the antioxidant effect of NAC that is responsible for the protection against copper ionophore-driven cell death [25]. To shed light on the role of GSH (and NAC), we preincubated HT-1080 cells with NAC (10 mM) or GSH (5 mM) followed by exposure for 24 h to Es/Cu. Both NAC and GSH abolished Es/Cu-induced cell death, and cytoplasmic vacuolization was also prevented (Fig. 8A,B). Furthermore, we found that the cellular GSH/GSSG ratio was diminished upon exposure to Es/Cu, while GSH levels were significantly restored not only by NAC, but also by the copper chelating agent, TTM and by the calpain inhibitor, PD150606 (Fig. 8C). Moreover, depletion of cellular GSH with buthionine sulfoximine (BSO), which inhibits the rate limiting step in GSH synthesis, did not affect cell viability per se, but we noted a dramatic enhancement of Cu/Es-induced cell death when compared to Es/Cu alone (Fig. 8D). Taken together, Es/Cu-induced cell death transpires with a depletion of GSH, and our findings show that GSH is required to protect cells from excess copper.

### Elesclomol compromises ubiquitin and proteasome homeostasis

**Fig. 9:**
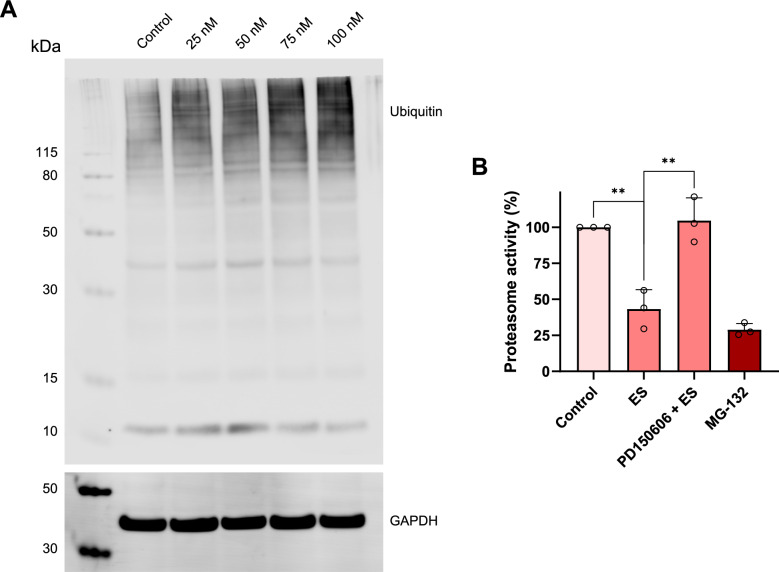
Elesclomol provokes proteotoxic stress. **A** Western blot of poly-ubiquitinated proteins in HT-1080 cells exposed to medium alone *versus* Es/Cu at the indicated concentrations for 24 h. GAPDH was used as a loading control. **B** 20S proteasome activity in HT-1080 cells exposed to Es/Cu (50 nM) in the presence or absence of the calpain inhibitor, PD150606 (100 µM). The proteasome inhibitor, MG-132 (500 nM) was included as a positive control. Data are shown as mean values ± S.D. (*n* = 3). **p < 0.01

Copper has been shown to trigger protein aggregation, which may explain the toxicity of copper, but it has also been speculated that protein aggregates may trap excess Cu, thereby preventing further cellular damage [26]. Indeed, copper is subject to tight control: it has been estimated that there is less than one free copper ion per cell [27], and copper ions appear to be coordinated to proteins or to ligands of low molecular mass such as GSH under normal conditions [28]. To understand whether Es/Cu evoked proteotoxic stress in our model, we performed western blotting of polyubiquitinated proteins. We observed a dose-dependent accumulation of polyubiquitinated proteins in HT-1080 cells exposed to Es/Cu for 24 h (Fig. 9A). The accumulation of polyubiquitinated proteins which are normally destined for degradation by the proteasome suggested that the proteasome might be compromised. Indeed, we observed a significant loss in activity of the 20S proteasome upon exposure to Es/Cu, comparable to that of the known proteasome inhibitor, MG-132 (Fig. 9B). Moreover, the calpain inhibitor, PD150606, restored proteasome activity. We previously provided evidence that copper-based nanoparticles, which undergo dissolution in lysosomes, trigger proteotoxic stress, and we obtained evidence of copper-induced protein aggregation using the PROTEOSTAT® aggresome detection reagent [29]. Aggresomes are inclusion bodies of misfolded proteins that form when the ubiquitin–proteasome protein degradation machinery is overwhelmed. We decided to use the same assay to study whether Es/Cu-induced cell death features aggresomes. However, while aggresomes were readily observed in cells exposed to MG-132, no inclusion bodies were detected in Es/Cu-exposed cells (Figure S8). Initially, we found this puzzling, as protein aggregation is believed to play a major role in copper-induced toxicity [30]. However, accumulation of misfolded proteins might occur in a different compartment not captured by the aggresome detection reagent. It is also conceivable that copper triggers protein precipitation through a mechanism independent of misfolding mechanisms, as suggested in a recent proteome-wide study of copper-induced protein precipitation [31].

### Proteomics profiling reveals a pronounced heat shock response

**Fig. 10:**
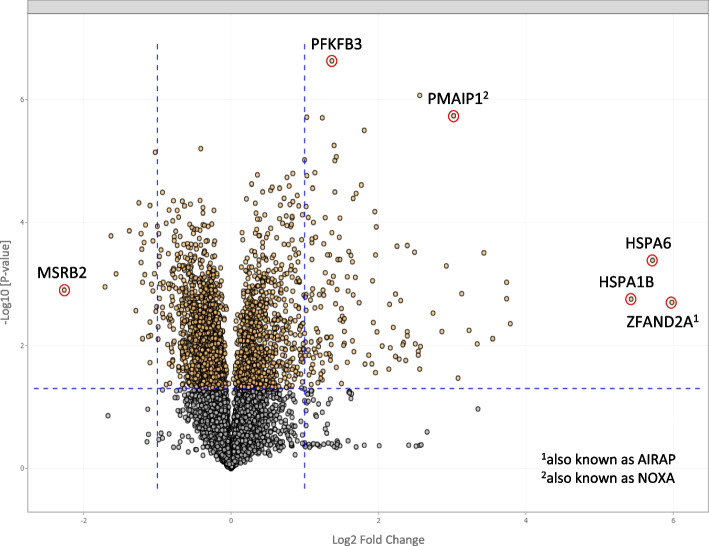
Volcano plot of differentially expressed proteins (DEPs) in HT-1080 cells exposed to Es/Cu *versus* control highlighting the most significantly up- and down-regulated proteins. The x-axis displays the log2 fold change of protein expression, where positive values indicate upregulated proteins and negative values correspond to downregulated proteins. The p-values are presented on a -log10 scale on the y-axis

To further unravel the cellular impact of Es/Cu, we performed proteomics profiling of HT-1080 cells exposed to Es/Cu (50 nM) for 24 h. The samples were analyzed by liquid chromatography with tandem mass spectrometry (LC–MS/MS) using an established protocol [32]. The volcano plot depicting the fold-change *versus* statistical significance of the differentially expressed proteins revealed a heat shock response characterized by the upregulation of a trio of heat shock proteins known as HSPA1B (43-fold upregulation), HSPA6 (53-fold upregulation), and ZFAND2A (63-fold upregulation) (Fig. 10). Remarkably, the same three proteins were found to be induced (ZFAND2A) or increased (HSPA1B and HSPA6) by copper in a previous study using a colorectal adenocarcinoma cell line [33], suggesting that these proteins may be considered a ‘hallmark’ of cellular copper excess. Indeed, it is worth noting that copper-based nanoparticles also trigger a conserved heat shock response with upregulation of HSP70 family members [34–36]. Moreover, ZFAND2A (also known as AIRAP) (arsenite-inducible RNA-associated protein) was previously shown to protect cells from arsenite toxicity [37, 38]. AIRAP encodes a proteasome-interacting protein, and previous work suggested that it serves to adapt the protein degradation machinery of the cell to counteract the proteotoxicity induced by the environmental toxin, arsenite [39]. We surmise that the strong induction of ZFAND2A by elesclomol may constitute a cytoprotective response, although further studies are required to formally prove this. Notwithstanding, ZFAND2A may be tentatively labeled as a ‘metal shock’ rather than heat shock protein. We also observed a significant upregulation of PMAIP1 (phorbol-12-myristate-13-acetate-induced protein 1) (also known as NOXA) (Fig. 10). NOXA (derived from the Latin word for ‘harm’ or ‘damage’) is a modulator of proteosome inhibition-induced cancer cell death [40], and a previous study demonstrated that dimethoxycurcumin, a methylated curcumin analog, triggered paraptosis with proteasome inhibition and upregulation of NOXA [41]. However, the upregulation of NOXA (eightfold) in our study was modest in comparison to the heat shock proteins. Canonical pathway analysis of the proteomics data was performed using the IPA software [42], and pathways related to protein ubiquitination and heat shock were significantly activated (Figure S9). Further analysis of the data using the STRING database for the identification of known and predicted protein–protein interactions [43] showed that upregulated proteins (n = 149) were enriched for pathways associated with protein misfolding (Figure S10) whereas downregulated proteins (n = 27) were enriched for the pathway associated with mitochondrial RNA metabolism (Figure S11). Overall, Es/Cu was shown to evoke proteotoxic stress accompanied by a heat shock response.

## Discussion

Copper ionophore-induced cell death has garnered considerable attention, yet our understanding of the mechanism underlying the demise of the cell is incomplete. Using the copper ionophore, elesclomol (Es), we show that copper-induced cell death is calpain-dependent with morphological attributes of paraptosis, a non-apoptotic, non-autophagic cell death first described 25 years ago by Bredesen and colleagues [11]. Several natural and synthetic compounds including metal complexes have been found to induce paraptosis [44, 45]. Indeed, the structurally unrelated copper-binding agents disulfiram and pyrazole-pyridine copper complexes were found to evoke paraptosis with simultaneous inhibition of caspase-3, the main ‘executioner’ in apoptotic cell death [46]. Paraptosis is characterized by extensive cytoplasmic vacuolization suggestive of alterations in cytoskeletal proteins [47], yet the cause of these drastic changes remains unknown, although oxidative stress and dysregulated calcium homeostasis have been implicated in this process [48]. We propose that calpain activation with cleavage of key substrates [49, 50] may contribute towards the remodeling of the cytoskeleton during paraptosis as PD150606 was found to prevent vacuolization and cell death in Es/Cu-exposed cells. Hypothetically, the dilation of the ER could also be linked to molecular ‘crowding’ due to the accumulation of misfolded proteins [51, 52]. However, the two mechanisms (i.e., biochemical and biophysical) are not mutually exclusive. Furthermore, we have demonstrated that copper-induced paraptosis is characterized by the perinuclear clustering of mitochondria. Perinuclear clustering of mitochondria is a common response to cellular stress [53], and is controlled by the cytoskeleton [54]. The current findings are summarized in schematic form in Figure S12, though it should be emphasized that some questions still remain. In particular, the mechanism whereby copper activates the calcium-dependent calpain(s) should be further scrutinized. Previous studies in yeast have shown that exposure to a surplus of copper is accompanied by an elevation in cytosolic calcium which was influenced not only by the concentration of copper but also by the overall oxidative status of the cell [55]. We also found that Es/Cu provoked a significant elevation of intracellular calcium alongside mitochondrial dysfunction. However, the cell permeable calcium chelating agent, BAPTA-AM failed to block cell death (data not shown). On the other hand, we found that DIDS, a non-selective inhibitor of VDAC1, blocked fodrin cleavage (a surrogate marker of calpain activation in the present study) and rescued cells from Es/Cu-induced cell death. This suggests the potential for crosstalk between the ER and mitochondria via VDAC1, a conduit for ions (including calcium) and small metabolites [22]. Further studies are required to pinpoint the role of VDAC1 in paraptosis.

Es and its complex with Cu(II) were initially thought to trigger apoptosis with the induction of oxidative stress, though the signaling pathway was not well defined [56, 57]. More recently, Tsvetkov et al. found that Es promoted a unique form of copper-dependent cell death that is subject to regulation by the mitochondrial ferredoxin, FDX1. [2, 4] This cell death, referred to as cuproptosis, is characterized by the aggregation of lipoylated proteins [8]. The question arises whether the Es/Cu-induced cell death reported herein is akin to cuproptosis. The answer is yes and no. First, while cuproptosis research remains in its infancy, it is clear that this cell death depends on mitochondrial metabolism. Hence, glycolysis-dependent cancer cells are less sensitive to cuproptosis than their mitochondrial respiration-dependent counterparts [8]. Indeed, when we replaced glucose with galactose to better mimic the conditions established by Tsvetkov et al., we found that cells became more susceptible to Es/Cu. Interestingly, cell death remained inhibitable but not preventable by PD150606. Moreover, FDX1 was recently identified as a mediator of cuproptosis directly regulating protein lipoylation through the binding of lipoyl synthase. [58] However, in our model, silencing of FDX1 did not impair Es/Cu-induced cell death. It has been noted by other investigators that intracellular copper delivery continues in the absence of FDX1, suggesting an alternative mechanism of copper delivery by Es [59]. We reasoned that STEAP3, an endosomal protein that serves not only as a ferrireductase but also as a cupric reductase [13], might play a role in the extramitochondrial reduction and release of copper. However, silencing of STEAP3 failed to block Es/Cu-induced cell death in our model. There are at least two possible solutions to this conundrum. First, another metalloreductase could be involved in the reduction of Cu(II) to Cu(I) [60]. Alternatively, the toxic entity is not Cu(I) but Cu(II), in which case reduction of Cu(II) may not be strictly required. However, TTM, which was found to block copper-induced cytoplasmic vacuolization and cell death in our model, is known to bind Cu(I) with high affinity [61]. Nevertheless, while Cu(I) has been implicated in the cytotoxicity of copper by virtue of interfering with cellular redox homeostasis leading to ROS production, both Cu(I) and Cu(II) are capable of inducing protein misfolding (directly or indirectly) [62]. Indeed, the cell’s ability to mount a heat shock response to counter copper-induced proteotoxic stress may be the key factor determining the outcome of copper toxicity [30]. In other words, proteotoxic stress rather than oxidative stress may be driving cell death. Moreover, we observed that Es/Cu-induced cell death was accompanied by a reduction of the ratio of reduced GSH to oxidized GSH (GSSG) while the depletion of cellular GSH using BSO further sensitized the HT-1080 cell line to Es/Cu. Indeed, it is well established that excess copper promotes GSH depletion [63, 64], and the elevation of mitochondrial copper levels along with the depletion of mitochondrial GSH was recently shown to promote radiotherapy-induced cuproptosis [65]. The protective effect of GSH has been attributed to the stabilization of copper in its cuprous form, compromising its ability to further free radical generating reactions [66]. Overall, GSH depletion emerges as a hallmark of different copper-induced cell death modalities along with the canonical heat shock response (discussed above). Finally, our results have demonstrated that Es/Cu-induced cell death is not confined to mitochondria, as we have demonstrated ER dilation, characteristic of paraptosis, along with perinuclear clustering of mitochondria, as well as proteasome inhibition and a heat shock response not restricted to the mitochondrial compartment, as evidenced by our proteomics analysis of HT-1080 cells. Thus, we can confirm that Es/Cu-induced cell death is non-apoptotic [2, 4], as it cannot be rescued by the pan-caspase inhibitor, zVAD-fmk, and we show that excess copper (delivered by elesclomol) evokes paraptosis in cancer cells under glucose-proficient conditions, and evidence is furnished that this cell death is calpain-dependent. Further studies are warranted to clarify how excess intracellular copper activates calpain and to determine the calpain isoform(s) involved in paraptosis.

### Experimental procedures

#### Chemicals

The Cu(II)-chelating malonohydrazide derivative, elesclomol (Es) was purchased from Sigma-Aldrich (Sweden) (cat. no. SML2651) (CAS no. 488832–69-5).

#### Cell culture

The human fibrosarcoma cell line, HT-1080, the human fibroblast cell line, BJ, and the human embryonic kidney cell line, HEK-293 T, were obtained from the American Type Culture Collection (ATCC). HT-1080 cells and BJ cells were grown in Minimum Essential Medium (MEM) (Gibco) while HEK-293 T cells were grown in Dulbecco’s modified Eagle medium (DMEM) (Gibco), supplemented with 10% fetal bovine serum (FBS) and 1% penicillin–streptomycin (Gibco). The cell lines were routinely tested for mycoplasma contamination using MycoAlert® detection kit (Lonza).

#### Cell viability and cytotoxicity assays

HT-1080 cells or BJ cells were seeded overnight (16 h) at 5,000 cells/well in a 96-well plate. Cells were then exposed to Es in the presence of 1 μM CuCl_2_ (Es-Cu) at the indicated concentrations. The alamarBlue™ assay was used to determine cell viability (metabolic capacity), while lactate dehydrogenase (LDH) release was determined to assess cytotoxicity [67] using the CytoTox 96® Non-Radioactive Cytotoxicity Assay (Promega). The absorbance values were measured using a Tecan Infinite® F200 plate reader (Männedorf, Switzerland). The percentage of metabolic capacity was calculated by normalizing values to the vehicle control, whereas the percentage of LDH release was calculated by normalizing to maximum LDH release values. To investigate the potential mechanism of cytotoxicity induced by Es/Cu, cells were pre-incubated for 2 h with inhibitors (Sigma), including the pan-caspase inhibitor zVAD-fmk, the ferroptosis inhibitor ferrostatin-1 (Fer-1), the necroptosis inhibitor necrostatin-1 (Nec-1), the autophagy inhibitor wortmannin, the copper chelating agent, tetrathiomolybdate (TTM), the selective calpain inhibitor PD-150606, the non-specific VDAC (voltage-dependent anion channel) inhibitor DIDS, the inositol trisphosphate (IP_3_) receptor inhibitor 2-aminoethoxydiphenylborate (2-APB), N-acetyl cysteine (NAC), or glutathione (GSH), followed by exposure to Es/Cu for 24 h.

#### GSH/GSSG ratio assay

The ratio of reduced GSH to oxidized GSH (GSSG) was determined using the GSH/GSSG-Glo™ Assay (Promega), as previously described [29]. Briefly, HT-1080 cells were seeded in a 96-well plate at a density of 5,000 cells per well one day prior to the experiment. The cells were then exposed as indicated for 16 h. Following exposure, supernatants were discarded, and 50 µL of either the glutathione lysis reagent or the oxidized glutathione lysis reagent was added to each well followed by shaking for 5 min at room temperature. Subsequently, 50 µL of the luciferin generation reagent was added to each well, followed by shaking and incubation at room temperature for 30 min. Then, 100 µL of the luciferin detection reagent was added to each well, and the plate was incubated for a further 15 min. Finally, the luminescence signal was measured on a Tecan Infinite® F200 plate reader (Männedorf, Switzerland).

#### Flow cytometry

Intracellular calcium levels were investigated using a fluorescent dye-based assay. To this end, high-affinity Fluo-4 AM (Invitrogen) and low-affinity Fluo-5N AM (Invitrogen) were used for the detection of low and high calcium levels, respectively, while Rhod-2 AM (Invitrogen) was used to detect mitochondrial calcium levels. HT-1080 cells were seeded in a 12-well plate at a density of 50,000 cells per well one day prior to the experiment. After treatment, the cells were stained with either Fluo-4, Fluo-5N, or Rhod-2 for 30 min. The cells were then washed with PBS, collected, centrifuged, and resuspended in Hanks’ balanced salt solution (HBSS). Fluorescence was measured using a BD Accuri™ C6 Plus flow cytometer (BD Biosciences). For the evaluation of mitochondrial function, cells were seeded in a 12-well plate overnight, followed by exposure as indicated for 16 h. The cells were then stained with MitoTracker™ Green FM (Invitrogen) and MitoTracker™ Deep Red FM (Invitrogen) or MitoSOX™ (Invitrogen) for 30 min at 37 °C. Thereafter, cells were collected by trypsinization, centrifuged for 5 min at 1,500 rpm, followed by washing with PBS and centrifuged again. Finally, cells were resuspended in PBS for acquisition using the BD Accuri™ C6 Plus flow cytometer (BD Biosciences). For mitochondrial membrane potential measurements, the tetramethylrhodamine, ethyl ester (TMRE) assay (Abcam) was used. Cells were exposed as indicated for 16 h. Exposure to CCCP (5 μM) for 10 min served as a depolarization control. The cells were then stained and collected for acquisition with the BD Accuri™ C6 Plus flow cytometer (BD Biosciences).

#### Fluorescence image cytometry

Apoptosis assays were performed using the advanced image cytometer NucleoCounter® NC-3000™ (ChemoMetec, Allerød, Denmark). HT-1080 cells seeded at 50,000 cells per well were exposed to etoposide (50 µM) (Sigma-Aldrich) for 16 h and subsequently stained according to the manufacturer’s instructions using (i) Hoechst 33,342, FLICA reagent (FAM), and propidium iodide PI, or (ii) Hoechst 33,342, annexin V-fluorescein isothiocyanate (FITC)-conjugated antibody and propidium iodide (PI). Cells were then analyzed for caspase activity and phosphatidylserine (PS) externalization, respectively, using the NucleoCounter® NC-3000™. Data analysis was performed using the NucleoView™ NC-3000™ software.

#### Transmission electron microscopy

TEM was performed to determine ultrastructural changes [68] in cells exposed to 50 nM Es/Cu *versus* DMSO (vehicle control) for 16 h. Cells were harvested by trypsinization and fixed with 2.5% glutaraldehyde in 0.1 M sodium phosphate buffer, pH 7.4, at room temperature for 1 h and further fixed overnight at 4 °C. After washing with 0.1 M phosphate buffer, samples were centrifuged and post-fixed in 2% osmium tetroxide in 0.1 M sodium phosphate buffer, pH 7.4, at 4 °C for 2 h. Cells were dehydrated using a gradient of ethanol followed by acetone and embedded in LX-112 resin (Ladd Research, Essex Junction, VT). Ultrathin Sects. (50–80 nm) prepared using a Leica EM UC6 microtome were contrasted with uranyl acetate followed by lead citrate, and examined using a Hitachi HT7700 120 kV TEM (Hitachi High-Tech, Tokyo, Japan). Digital images were captured by using a Veleta 2 k × 2 k side-mounted TEM CCD camera (Olympus Soft Imaging Solutions, Germany).

#### Confocal microscopy

HT-1080 cells were seeded overnight at a density of 50,000 cells per well on poly-L-lysine-coated coverslips in 12-well plates. The following day, cells were exposed to 50 nM of Es/Cu for 24 h. Then, the cells were washed three times with PBS, fixed with 4% paraformaldehyde for 15 min, permeabilized with 0.1% Triton-X 100 for 15 min, and blocked with 3% BSA for 1 h, followed by incubation with anti-calreticulin antibody (ab22683; Abcam) (ER marker) for 1 h at room temperature. The coverslips were then incubated with anti-mouse-IgG conjugated to Alexa Fluor™ 488 (Invitrogen) for 1 h at room temperature. The cells were counterstained for 15 min with Hoechst 33342 (0.5 μg/mL) (ImmunoChemistry Technologies) to visualize cell nuclei. Alternatively, cells were stained with MitoTracker™ Green FM (Invitrogen), MitoTracker™ Deep Red FM (Invitrogen), or F-actin-binding Phalloidin-iFluor™ 647 (Abcam). Finally, the samples were mounted with Prolong™ Diamond Antifade Mountant (Invitrogen) and imaged on a Leica Stellaris 5 X confocal microscope (Leica).

#### Gene silencing

The RNAi consortium (TRC) lentiviral human *FDX1* and *STEAP3* shRNA plasmids TRCN0000056594 (sh-*FDX1*#1), TRCN0000056597 (sh-*FDX1*#2), TRCN0000292090 (sh-*STEAP3*#1), and TRCN0000292092 (sh-*STEAP3*#2) were purchased from Dharmacon. To generate lentiviral particles, HEK293T cells were co-transfected with MISSION® Lentiviral Packaging Mix (SHP001, Sigma-Aldrich), along with either MISSION® TRC2 pLKO.5-puro non-target shRNA control plasmid (sh-NT; SHC216, Sigma-Aldrich) and sh-*FDX1*#1, sh-*FDX1*#2, sh-*STEAP3*#1, or sh-*STEAP3*#2. After 24 h, supernatants containing viral particles were collected and filtered through a 0.45 μm PES sterile syringe filter (VWR). Polybrene (Sigma-Aldrich) was added to the lentiviral preparation at a concentration of 8 μg/mL and then used to infect cells. After 24 h of infection, cells were selected with puromycin (Gibco) for 48 h.

#### Western blot

For protein detection, cells were washed twice with cold phosphate-buffered saline (PBS) and harvested in Pierce™ RIPA buffer (Thermo Scientific) with freshly added protease inhibitor cocktail (Thermo Scientific). Then, cell lysates were centrifuged at 12,000 rpm for 10 min at 4 °C, and the supernatants were collected for immunoblotting [69]. The total protein concentration was determined using the Pierce™ Bradford Plus Protein Assay Reagent (Thermo Scientific), and 30 μg of protein per sample was loaded onto a pre-cast NuPAGE™ Bis–Tris Mini Protein Gels, 4–12% (Invitrogen). Proteins were then transferred onto Immobilon®-FL PVDF Membrane (Millipore). The membranes were blocked in Intercept® (TBS) Blocking Buffer (LI-COR Biosciences) at room temperature for 1 h, and membranes were incubated with primary antibodies in Intercept® T20 (TBS) Antibody Diluent (LI-COR Biosciences) at 4 °C overnight. The primary antibodies used were rabbit anti-STEAP3 monoclonal antibody (Abcam), rabbit anti-FDX1 polyclonal antibody (Proteintech), mouse anti-α-fodrin monoclonal antibody (Sigma-Aldrich), rabbit anti-ubiquitin polyclonal antibody (Invitrogen), and rabbit anti-calpastatin polyclonal antibody (Cell Signaling Technology), while a mouse anti-GAPDH monoclonal antibody (Invitrogen) was used as loading control. After washing, the membranes were incubated with IRDye® 800CW-labeled goat anti-rabbit IgG secondary antibody, or IRDye® 680RD-labeled goat anti-mouse IgG secondary antibody (LI-COR Biosciences). The membranes were scanned and imaged by using the Odyssey® CLx Imager imaging system (LI-COR Biosciences).

#### Proteasome activity assay

Proteasome activity in HT-1080 cells was determined using the fluorometric Proteasome 20S Activity Assay Kit (Sigma-Aldrich), as previously described [29]. Briefly, cells were seeded at a density of 5,000 cells/well in a 96-well plate. The following day, cells were exposed as indicated for 24 h. MG-132 (500 nM) (Sigma) was included as a positive control. Following exposure, the fluorogenic substrate LLVY-R110 was added, and samples were incubated for 2 h at 37 °C. Fluorescence values were recorded by using a Tecan Infinite® F200 plate reader (Männedorf, Switzerland).

#### Protein aggregation assay

To detect aggregated proteins, HT-1080 cells were seeded overnight at a density of 50,000 cells per well on poly-L-lysine-coated coverslips in a 12-well plate. The following day, cells were exposed to 50 nM Es-Cu or 500 nM MG132 for 16 h. Then, cells were washed three times with PBS, fixed with 4% paraformaldehyde for 15 min, and permeabilized with 0.1% Triton-X 100 for 15 min, followed by staining for 30 min at room temperature using the PROTEOSTAT® Aggresome Detection Kit (Enzo Life Sciences). The samples were counterstained with 0.5 μg/mL Hoechst 33342. Then, the samples were washed twice with PBS, and coverslips were mounted on glass slides with Prolong™ Diamond Antifade Mountant (Invitrogen), and imaged by using the Leica Stellaris 5 X confocal microscope (Leica).

#### NanoLC-MS/MS analysis

HT-1080 cells were exposed to 50 nM Es/Cu or to vehicle alone (0.5% DMSO) for 24 h. The experiment was performed in triplicates. Then, cells were washed with cold PBS, collected by scraping, and stored at −80°C for further analysis. The samples were processed as previously described [32]. Briefly, cells were lysed by subjecting them to freeze–thaw cycles in liquid nitrogen 5 times, followed by probe sonication for 1 min (10 cycles of 3 s at 30% amplitude with 3 s of pause between them) on ice. After centrifugation for debris removal at 14,000 at 4 °C for 30 min, supernatants were collected. The total protein concentration of extracted samples was measured using micro-BCA assay and 50 μg of protein was processed by 8 mM dithiothreitol (Sigma) reduction, and 25 mM iodoacetamide (Sigma) alkylation. Then, samples were precipitated using cold acetone at −20 °C overnight. Samples were resuspended in EPPS (4-(2-hydroxyethyl)−1-piperazinepropanesulfonic acid) buffer, 8 M urea, pH 8.0, diluted down to 4 M urea and digested by LysC, then diluted down to 1 M urea and digested with trypsin. Each digest was labeled using TMTpro 18-plex technology (Thermo Fischer) and a final multiplex sample was cleaned by Sep-Pack C18 column (Waters). The final sample was fractionated off-line by capillary reversed phase chromatography at pH 10 into 48 fractions, and each of them was then analyzed by high-resolution nLC-ESI–MS/MS (nanoscale liquid chromatography-electrospray ionisation-tandem mass spectrometry) using an Orbitrap Exploris™ 480 mass spectrometer (Thermo Fisher Scientific) equipped with an EASY ElectroSpray source and connected to an Ultimate 3000 nanoflow UPLC system, as previously described [32].

#### Proteomics data analysis

Proteome Discoverer 3.1 (Thermo Scientific) was utilized for the database search and quantification against the Uniprot *Homo sapiens* (Human) protein database (UP000005640). We employed a 1% false discovery rate (FDR) as a filter both at the protein and peptide level. Only proteins with at least two unique peptides were included in the quantitative analysis. Proteins with missing values were eliminated. The quantified abundance of each protein in each sample labeled with a different tandem mass tag (TMT) was normalized to the total intensity of all proteins in that sample. For each protein in each replicate, the normalized protein abundance was divided by the average abundance of that protein in the vehicle control replicates. The average ratio across replicates compared to the vehicle control was calculated, and the log_2_ values of these ratios were determined. Student's t-test (two sided with assumption of unequal variance) was employed to calculate the p-value, assuming a non-zero ratio. Proteins with *p* < 0.05 and log_2_ (fold-change) > 1 were considered differentially expressed proteins (DEPs), and visualized by plotting the log_2_ ratio on the x-axis and the corresponding -log_10_ p-value on the y-axis. DEPs were divided into upregulated and downregulated proteins, and protein–protein interaction (PPI) and functional enrichment analysis were performed using version 12.0 of the STRING database (https://www.string-db.org.) [43] The Markov cluster (MCL) algorithm was applied for clustering of proteins in PPI networks, with the inflation parameter set to 4. Canonical signaling pathway analysis of the proteomics data was performed using the QIAGEN IPA software (Redwood City, CA) (.https://digitalinsights.qiagen.com/IPA) [42].

#### Statistical analysis

The results shown are derived from at least three independent experiments, and presented as mean values ± S.D. Data were analyzed using GraphPad Prism 8.0. Two-tailed unpaired Student’s *t*-test was used to assess differences between groups, and *p* < 0.05 was considered to be statistically significant.

## Supplementary Information

Supplementary Material 1.

## Data Availability

The data supporting the findings are contained within the article, or can be requested from the corresponding author. The mass spectrometry data have been deposited to the ProteomeXchange consortium via the PRIDE partner repository [70] (accession no.: PXD067483).

## Abbreviations

2-APB: 2-Aminoethoxydiphenylborate
BSO: Buthionine sulfoximine
CCCP: Carbonyl cyanide *m*-chlorophenyl hydrazine
DEPs: Differentially expressed proteins
DIDS: 4,4’-Diisothiocyanatostilbene-2,2’-disulfonate
ER: Endoplasmic reticulum
Es: Elesclomol
FDX1: Ferredoxin 1
GSH: Glutathione
GSSG: Glutathione disulphide
IP_3_: Inositol trisphosphate
LC–MS/MS: Liquid chromatography with tandem mass spectrometry
LDH: Lactate dehydrogenase
MAMs: Mitochondria-associated membranes
NAC: N-acetylcysteine
SERCA: Sarcoplasmic/endoplasmic reticulum calcium-ATPase
STEAP3: Six transmembrane epithelial antigen of prostate 3
TCA: Tricarboxylic acid
TRC: The RNAi consortium
TMRE: Tetramethylrhodamine, ethyl ester
TTM: Tetrathiomolybdate
VDAC1: Voltage-dependent anion-selective channel 1
zVAD-fmk: Carbobenzoxy-Val-Ala-Asp-fluoromethylketone
