# Supplementary material for: Copper ionophore elicits calpain-dependent paraptosis coincident with proteotoxic stress
Source: Cell Commun Signal. 2025 Dec 12;24:47. doi: 10.1186/s12964-025-02558-5 (PMC12837948; doi:10.1186/s12964-025-02558-5)
Supplement: Supplementary file 1 — Supplementary Material 1. [file 12964_2025_2558_MOESM1_ESM.pdf]

Supporting Information

Copper ionophore elicits calpain-dependent paraptosis coincident with proteotoxic stress

Apiwit Sae-Fung[1, 2], and Bengt Fadeel[1,*]

[1]Division of Molecular Toxicology, Institute of Environmental Medicine, Karolinska Institutet, Stockholm, Sweden; [2]Graduate Program in Clinical Biochemistry and Molecular Medicine, Department of Clinical Chemistry, Faculty of Allied Health Sciences, Chulalongkorn University, Bangkok, Thailand. *E-mail: bengt.fadeel@ki.se

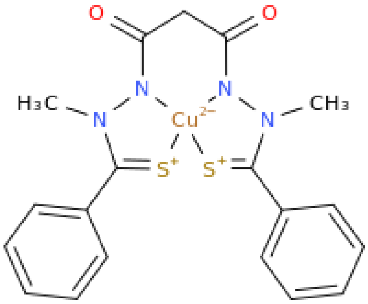

Figure S1. The copper coordination complex formed by elesclomol (Es) through binding to Cu(II) *via* the thiocarbonyl and hydrazino groups. From: PubChem (https://pubchem.ncbi.nlm.nih.gov) (Kim et al., Nucleic Acids Res. 2025;53(D1):D1516-D1525).

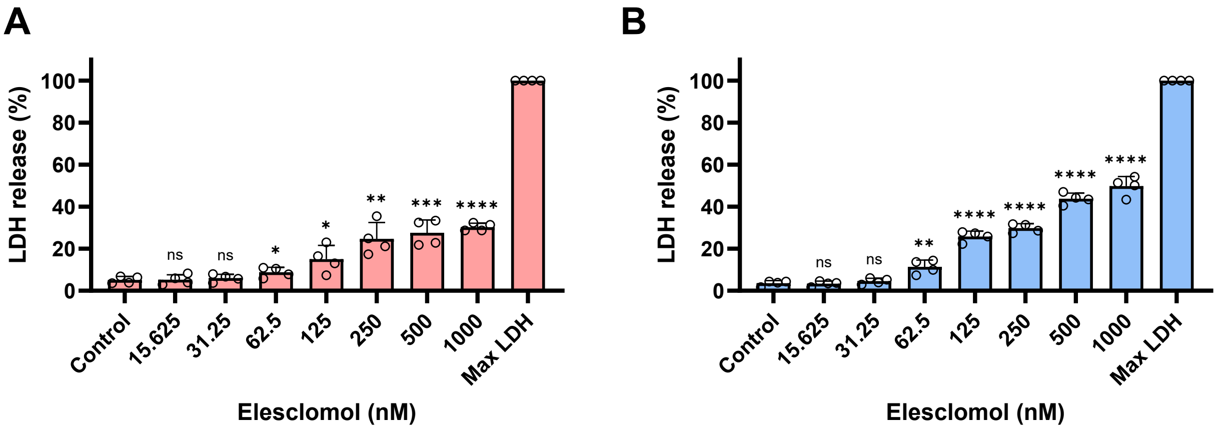

Figure S2. The fibrosarcoma cell line HT-1080 (A) and normal fibroblast cell line BJ (B) were exposed to Es/Cu at the indicated concentrations for 24 h and cell viability was determined by using the LDH release assay while metabolic activity was determined using the Alamar Blue assay (Figure 1A).

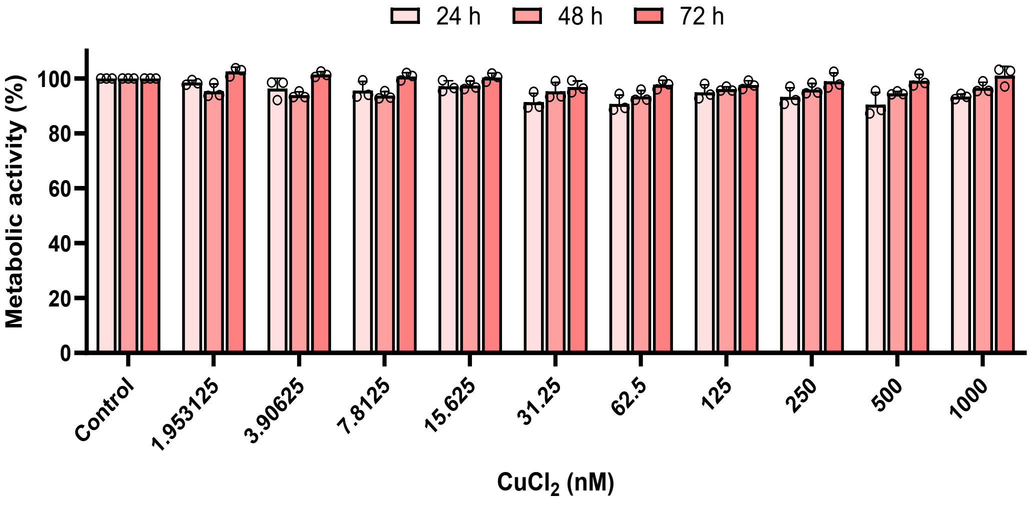

Figure S3. The fibrosarcoma cell line HT-1080 was exposed to $CuCl_2$ alone for 24, 48, and 72 h, and metabolic activity was determined using the Alamar Blue assay.

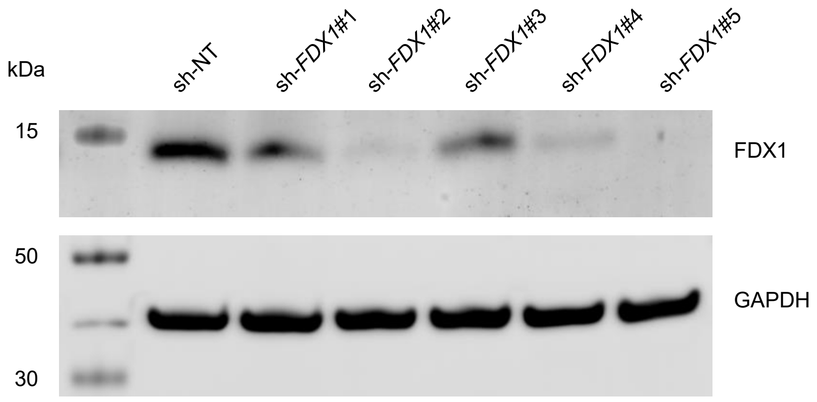

Figure S4. Gene silencing of *FDX1*. The expression of FDX1 in HT-1080 cells transduced with non-targeting (NT) shRNA and five different *FDX1*-targeting shRNAs was determined by Western blot. GAPDH was used as a loading control.

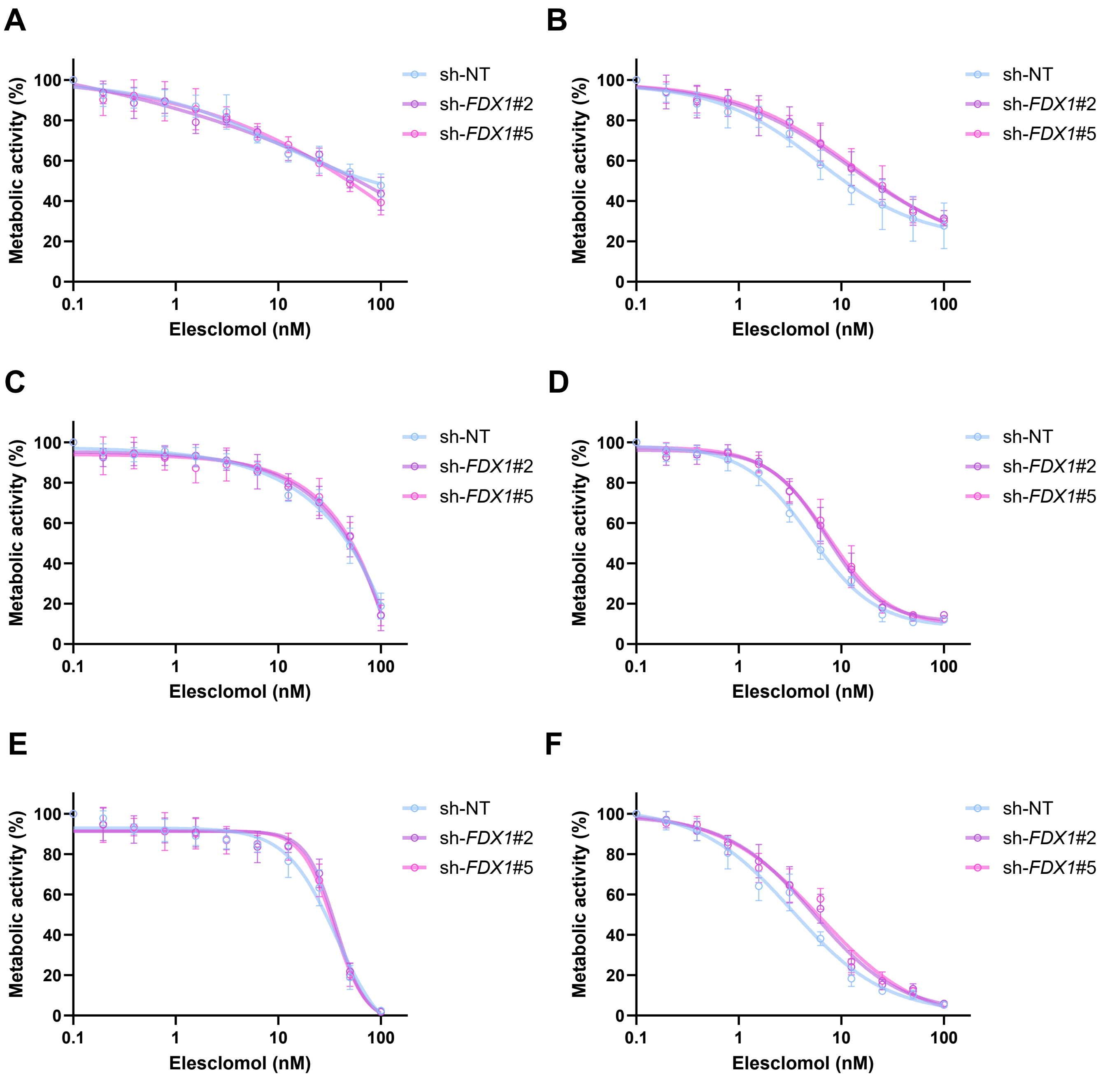

Figure S5. Metabolic capacity of HT-1080 cells with or without silencing of FDX1 were exposed to Es/Cu for 24 h (A, B), 48 h (C, D), and 72 h (E, F). Cells were maintained in medium supplemented with glucose (A, C, E) or galactose (B, D, F). Data shown are mean values ± S.D. (n=3).

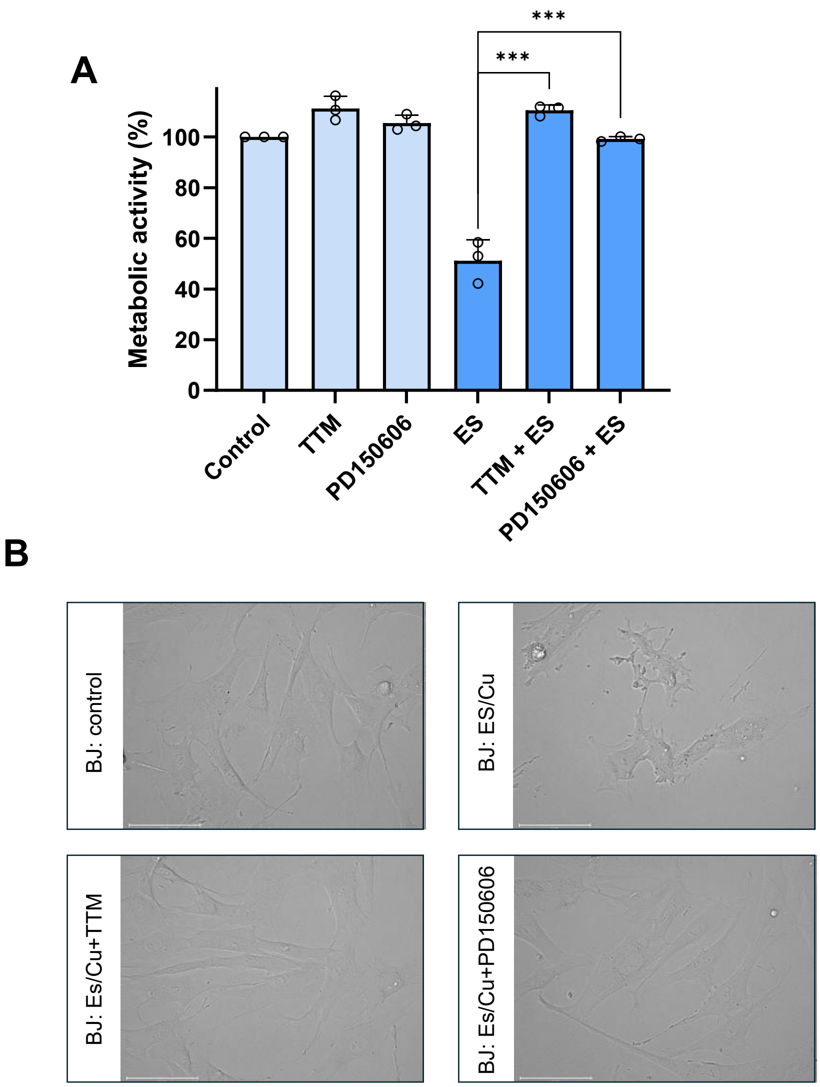

Figure S6. (A) BJ cells were exposed to Es/Cu for 24 h in the presence and absence of the copper chelating compound TTM (10 μM) or the calpain inhibitor PD150606 (100 μM) and metabolic capacity was determined by using the Alamar Blue assay. Data are shown as mean values ± S.D. (n=3). ****p* < 0,005. (B) BJ cells exposed as detailed above were visualized under the light microscope. The morphological changes elicited by Es/Cu were blocked by TTM and PD150606.

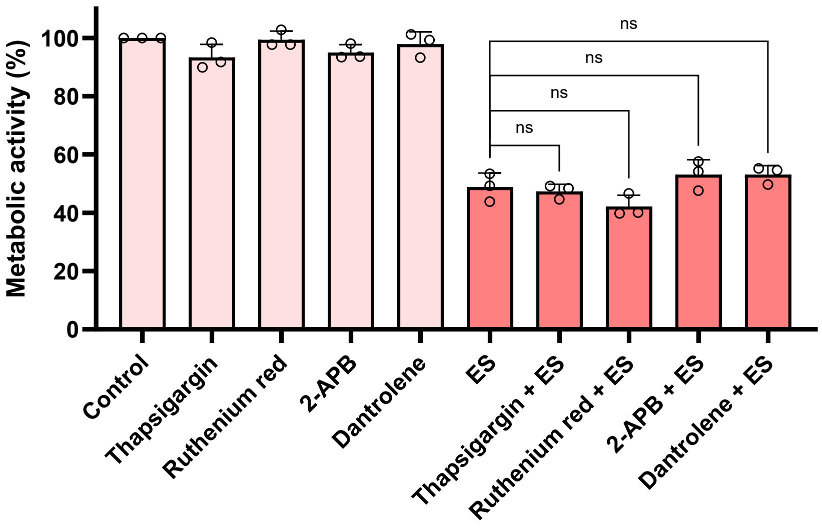

Figure S7. Exploring the role of intracellular calcium stores using pharmacological inhibitors. HT-1080 cells were exposed to Es/Cu (50 nM) for 24 h in the presence or absence of thapsigargin (40 nM), ruthenium red (5 µM), 2-APB (10 µM), or dantrolene (50 КM), and metabolic activity was determined using the Alamar Blue assay. Thapsigargin inhibits the sarcoplasmic/endoplasmic reticulum calcium-ATPase pump, ruthenium red and dantrolene block ryanodine receptors, 2-APB is an inositol trisphosphate (IP_3) receptor antagonist. For results using DIDS, an inhibitor of VDAC1 oligomerization, refer to Figure 6. Data are shown as mean values ± S.D. (n=3). ns, not significant.

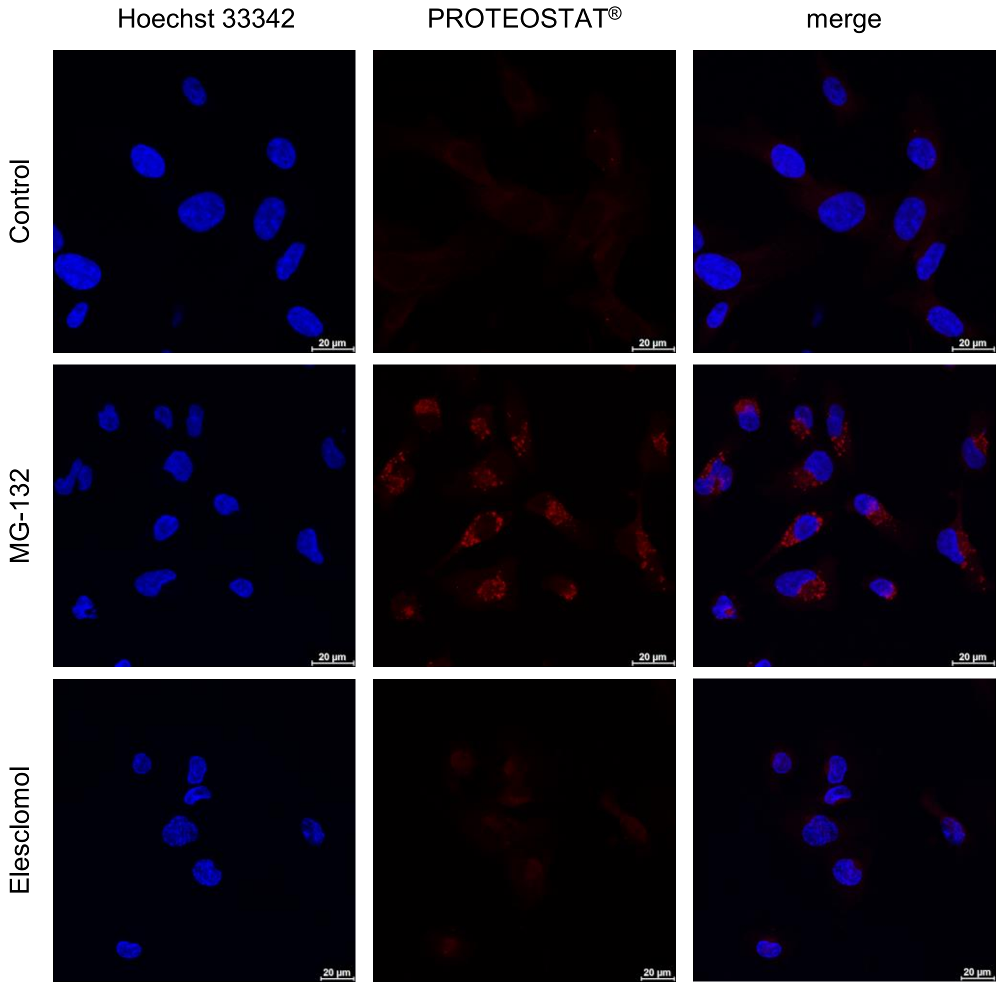

Figure S8. HT-1080 cells were exposed to MG-132 (500 nM) or Es-Cu (50 nM) for 16 h, and protein aggregation (i.e., aggresome formation due to the aggregation of misfolded proteins) was determined using the PROTEOSTAT® reagent (red). Cell nuclei were counterstained with Hoechst (blue).

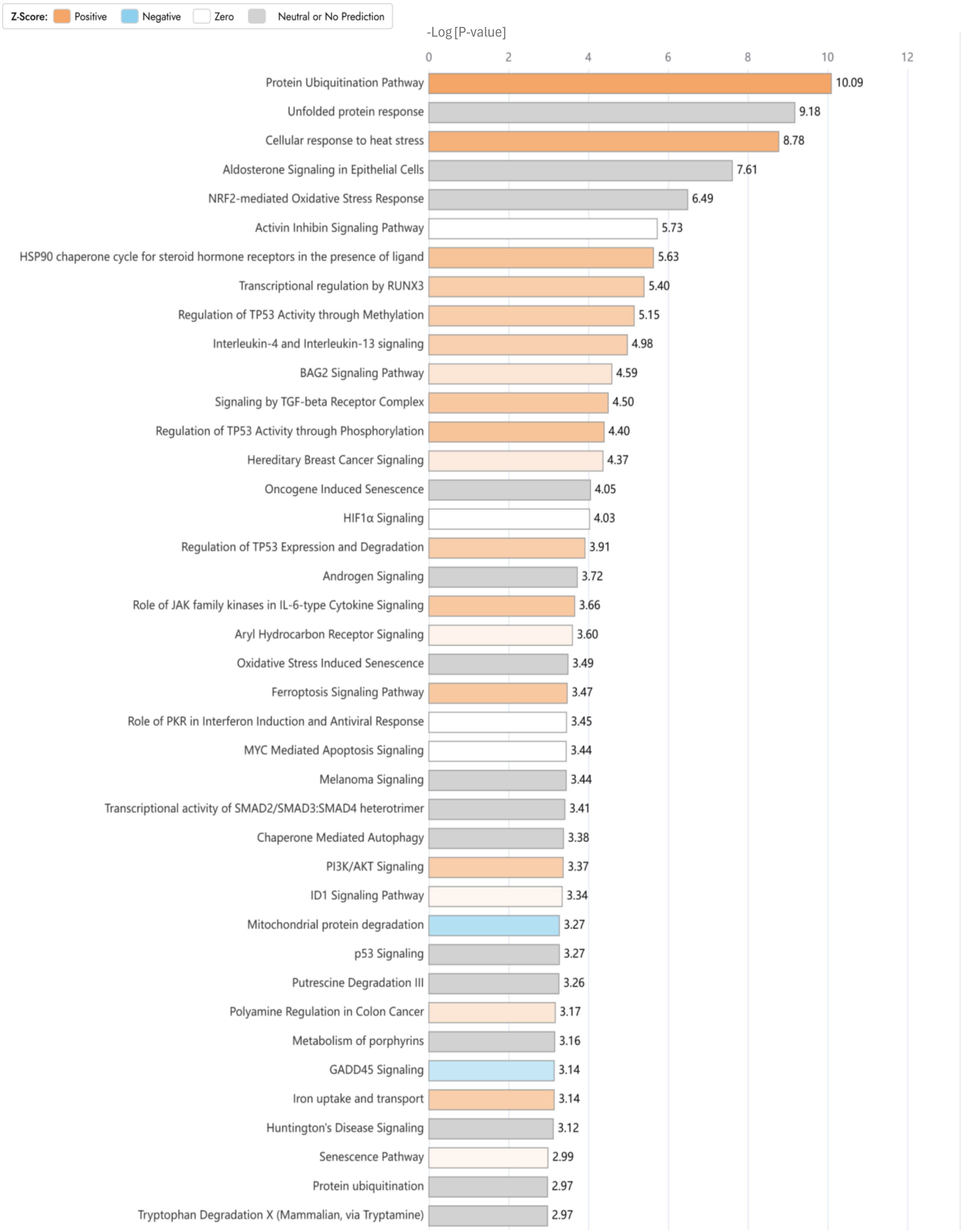

Figure S9. Canonical pathway analysis of differentially expressed proteins in Es/Cu-exposed samples. Data were analyzed by using QIAGEN IPA (https://digitalinsights.qiagen.com/IPA). The bar chart represents the top-40 signaling and metabolic pathways. A positive z-score (orange) denotes pathway activation, and a negative z-score (blue) denotes pathway inhibition.

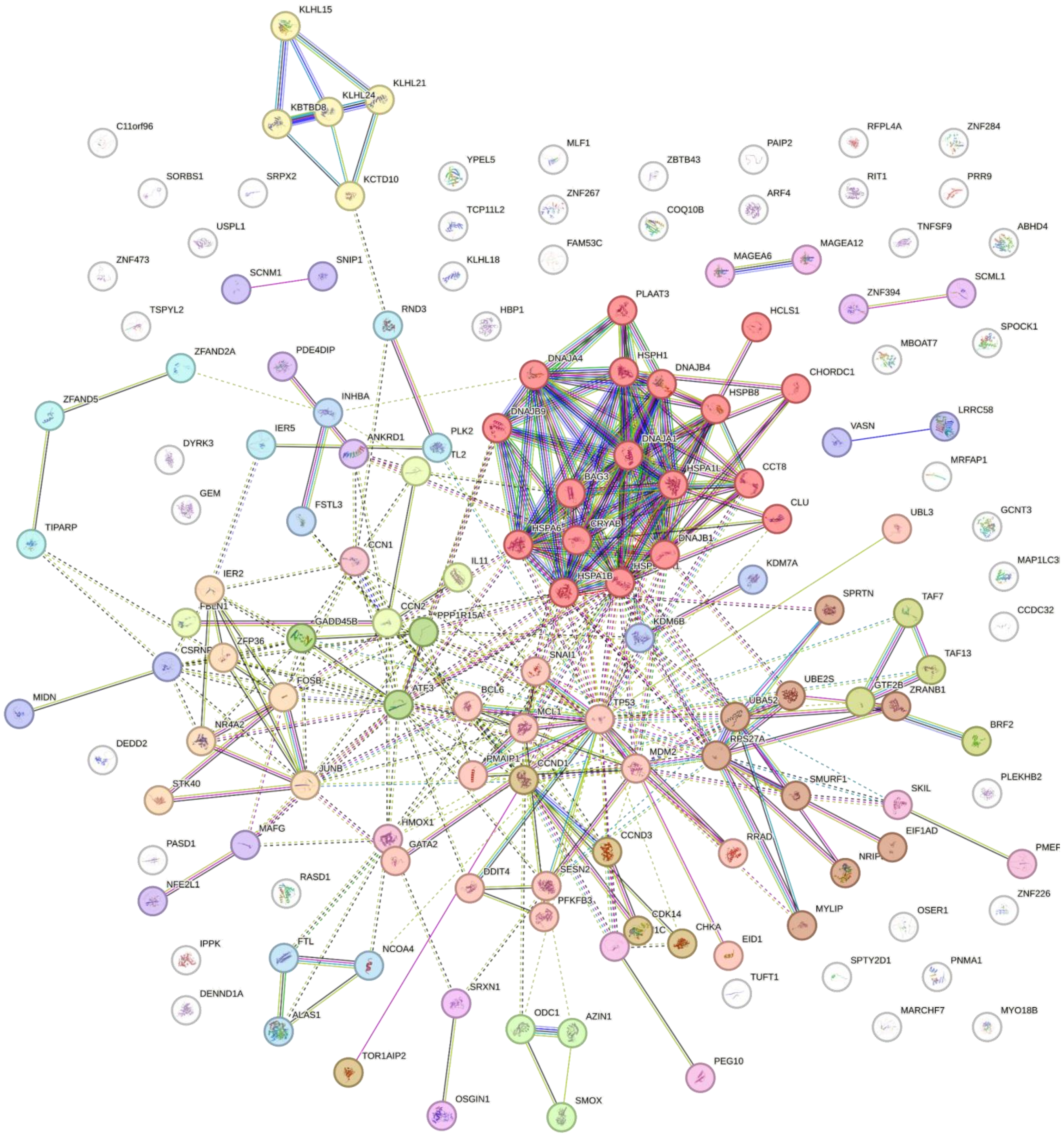

Clusters

color	cluster Id	gene count	description
●	Cluster 1	18	1. Response to topologically incorrect protein<br>2. Unfolded protein binding
●	Cluster 2	13	1. Signal transduction by p53 class mediator<br>2. p53 signaling pathway
●	Cluster 6	5	Cul3-RING ubiquitin ligase complex
●	Cluster 11	3	AN1-like Zinc finger

Figure S10. STRING analysis of all the significantly upregulated proteins (n=149) in HT-1080 cells exposed to Es/Cu (50 nM). The clusters are color-coded and selected clusters are clarified below the graph.

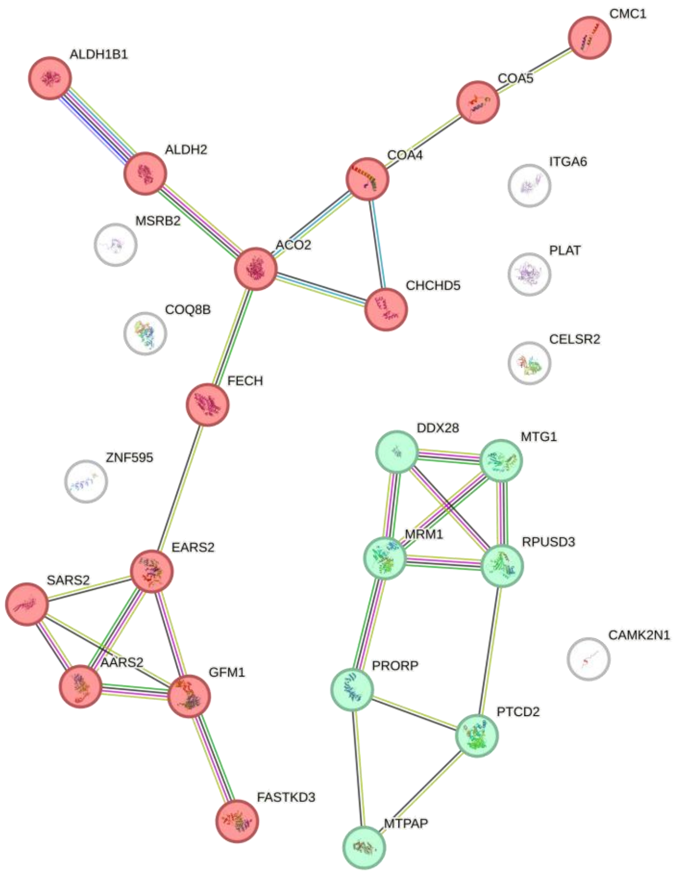

Clusters

color	cluster Id	gene count	description
● (red)	Cluster 1	13	Mitochondrial RNA metabolism
● (green)	Cluster 2	7	rRNA modification in the mitochondrion, and Regulation of mitochondrial translation

Figure S11. STRING analysis of all the significantly downregulated proteins (n=27) in HT-1080 cells exposed to Es/Cu (50 nM). The description of the two identified clusters is provided below the graph. The analysis was performed using the STRING database, version 12.0 (https://www.string-db.org).

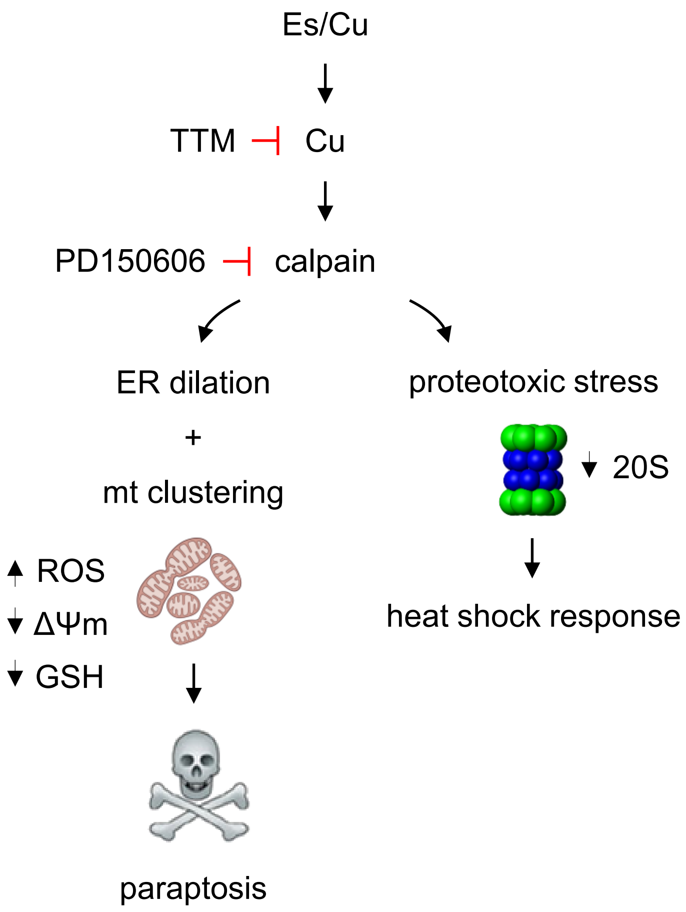

Figure S12. Current model of Es/Cu-triggered cell death. The key finding in the present study is that the copper ionophore elesclomol (Es) triggers calpain-dependent cell death with morphological attributes of *paraptosis* (ER dilation) along with perinuclear mitochondrial clustering albeit in the absence of the chromatin compaction that is typically seen in cells undergoing apoptosis. Es/Cu was also shown to trigger calcium elevation and mitochondrial dysfunction, and DIDS, a non-selective inhibitor of VDAC1, rescued cells from Es/Cu, suggesting the crosstalk between ER and mitochondria (not shown). Furthermore, we have provided evidence of proteasome inhibition in cells exposed to Es/Cu. This could potentially be due to protein misfolding leading to proteasomal insufficiency and/or to a direct inhibitory effect of copper on the proteasome. We could also confirm a heat shock response in exposed cells.
